# Consumer Health Informatics to Advance Precision Prevention

**DOI:** 10.1055/s-0044-1800735

**Published:** 2025-04-08

**Authors:** Oliver J. Canfell, Leanna Woods, Deborah Robins, Clair Sullivan

**Affiliations:** 1 Department of Nutritional Sciences, Faculty of Life Sciences and Medicine, King's College London, London SE1 9NH, United Kingdom; 2 School of Public Health, Faculty of Medicine, The University of Queensland, Herston QLD 4006, Australia; 3 Queensland Digital Health Centre, Centre for Health Services Research, Faculty of Medicine, The University of Queensland, Herston QLD 4006, Australia; 4 Consumer co-author, Queensland, Australia; 5 Metro North Hospital and Health Service, Queensland Health, Herston QLD 4006, Australia

**Keywords:** Consumer health informatics, Consumer health information, Medical informatics, eHealth, Chronic disease, Preventive medicine, Patients, Precision medicine, Health care professionals, Population health

## Abstract

**Objective** : Consumer health informatics (CHI) has the potential to disrupt traditional but unsustainable break-fix models of healthcare and catalyse precision prevention of chronic disease – a preventable global burden. This perspective article reviewed how consumer health informatics can advance precision prevention across four research and practice areas: (1) public health policy and practice (2) individualised disease risk assessment (3) early detection and monitoring of disease (4) tailored intervention of modifiable health determinants.

**Methods** : We review and narratively synthesise methods and published recent (2018 onwards) research evidence of interventional studies of consumer health informatics for precision prevention. An analysis of research trends, ethical considerations, and future directions is presented as a guide for consumers, researchers, and practitioners to collectively prioritise advancing two interlinked fields towards high-quality evidence generation to support practice translation. A health consumer co-author provided critical review at all stages of manuscript preparation, moderating the allied health, medical and nursing researcher perspectives represented in the authorship team.

**Results** : Precision prevention of chronic disease is enabled by consumer health informatics methods and interventions in population health surveillance using real-world data (e.g., genomics) (public health policy and practice), disease prognosis (regression modelling, machine learning) (individualized disease risk assessment), wearable devices and mobile health (mHealth) applications that generate digital phenotypes (early detection and monitoring), and targeted behaviour change interventions based upon personalized risk algorithms (tailored intervention of modifiable health determinants). In our disease case studies, there was mixed evidence for the effectiveness of consumer health informatics to improve risk-stratified or behavioural prevention-related health outcomes. Research trends comprise both consumer-centred and healthcare-centred innovations, with emphasis on inclusive design methodologies, social licence of health data use, and federated learning to preserve data sovereignty and maximise cross-jurisdictional analytical power.

**Conclusions** : Together, CHI and precision prevention represent a potential future vanguard in shifting from traditional and inefficient break-fix to predict-prevent models of healthcare. Meaningful researcher, practitioner, and consumer partnerships must focus on generating high-quality evidence from methodologically robust study designs to support consumer health informatics as a core enabler of precision prevention.

## 1. Introduction

Consumer health informatics (CHI) is a rapidly growing field that draws from transdisciplinary roots across the healthcare continuum in health promotion, health education, public health informatics, nursing informatics, information systems, psychology, and communication science, as examples[ 1 ]. CHI integrates diverse real-world data and digital technologies centred around consumers, including wearables, mobile health (mHealth), artificial intelligence (AI), genomics, electronic health records (EHRs), and the Internet of Things (IoT) to facilitate real-time monitoring, early disease detection and timely intervention. Research and practice momentum in CHI is motivated by the opportunity to decentralise healthcare delivery, encourage self-monitoring and management of long-term chronic conditions across settings outside of acute care ( *e.g.* , home, community), and improve health care professionals' access to routinely collected and (near) real-time measured consumer health data.

The consumer voice is starting to be integrated into designing, implementing, and evaluating new healthcare interventions and services; however, there is opportunity for growth [ 2 ]. A recent position paper from the American Medical Informatics Association (AMIA) [ 3 ] that reported a practice analysis of clinical informatics included numerous tasks that are patient-centred but not yet ubiquitous in care delivery and practitioner skill, such as social determinants of health, use of patient-generated healthcare data, and consumer health applications ( *e.g.* , patient portals, mHealth apps, disease management, heath behaviour monitoring). These applications are being developed rapidly yet their uptake and use in healthcare is inequitable across jurisdictions, and dependent upon decision-making at the individual health service level.

In the 2016 edition of the Yearbook of Medical Informatics, Demiris offered a comprehensive overview of the past, present, and future of CHI and predicted that precision medicine would become a key tenet in a 25-year future vision of the field [ 4 ]. A bibliometric study of the evolutionary overview of CHI between 1999-2019 proposed electronic medical records, technology, and natural language processing as emerging themes in future consumer health research [ 5 ]. Measuring individual variability in consumer health indicators to inform self-management and medical decision-making can now be powered by large-scale biological datasets ( *e.g.* , UK Biobank), advanced AI (machine learning) techniques ( *e.g.* , deep learning, natural language processing) to identify undiscovered patterns within datasets and ‘omics ( *e.g.* , proteomics, transcriptomics, metabolomics, genomics) [ 4 ].

The traditional collection and analysis of this precise data has been disease-centred and conducted within tightly controlled clinical trial or cohort settings [ 6 ]. Social, environmental, and behavioural health indicators explain ~70% of health variance; yet, informatics still focuses on data collection at the point-of-care to inform treatment decision-making, rather than at the consumer and community level to inform health promotion and prevention decisions [ 7 , 8 ]. CHI is a research discipline that can bridge this gap and in doing so, can forge a new era of ‘precision prevention’.

Precision prevention has suffered from diverse definitions in the literature and lack of a united empirical direction to generate evidence for its effectiveness. For this survey, we offer a simple and synthesised definition of precision prevention across four independent sources of literature [ 6 , 9 10 11 ] (Box 1).

**Figure 1:**
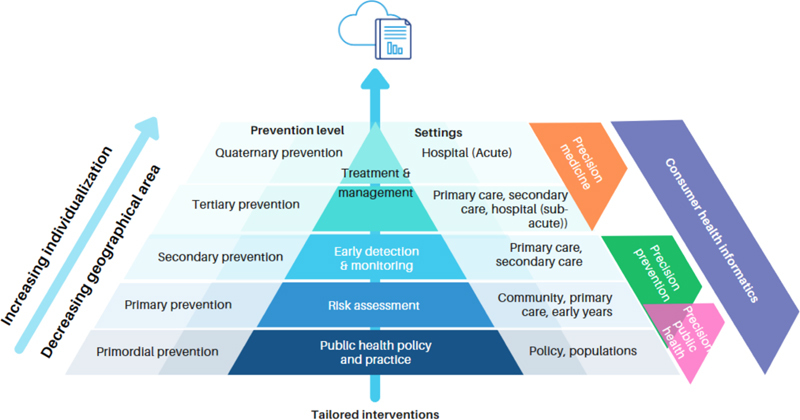
Consumer health informatics and precision models of care – a research and practice pyramid. Informed by and adapted from Canfell et al.,[ 6 ].

A significant research gap remains in understanding how CHI could enable targeted disease prevention [ 4 ]. CHI may act as an enabler to achieving precision prevention across three prevention levels: primordial (addressing social determinants to prevent risk factor development); primary (to prevent the onset of disease); and secondary (to detect and monitor disease with early diagnosis) [ 12 ] ( Figure 1 ). Tertiary prevention (disease management) and quaternary prevention (iatrogenic disease)[ 12 ] fall under the umbrella of precision medicine and are outside the scope of precision prevention ( *e.g.* , self-management of type 2 diabetes via an mHealth app, genomics testing to inform precision cancer treatment). Given the relative nascency of CHI for precision prevention of chronic disease, this perspective paper provides a narrative overview of contemporary (2018 onwards) methods and interventions related to the intersection of these two fields across four research and practice areas:

- Public health policy and practice (primordial prevention);
- Individualised disease risk assessment (primary prevention);
- Early detection and monitoring of disease (secondary prevention);
- Tailored intervention of modifiable health determinants (primordial, primary, and secondary prevention).

The survey concludes by discussing research trends, ethical considerations, and future directions to advance the two disciplines. Our intent is to provide a preliminary evidence map of the intersection between CHI and precision prevention that could inform a future systematic review and meta-analysis of intervention effectiveness to improve prevention-related health outcomes.

## 2. Methods

We reviewed and integrated literature on methods and interventions that linked CHI with precision prevention. Medline and Embase were searched for relevant articles published from 2018 onwards to reflect the recent emergence of the two disciplines that could act as case studies of the topic. MeSH terms and key words relating to CHI and precision prevention were used. Findings from selected articles were narratively summarised and deductively applied to the four-step continuum of research and practice in CHI for precision prevention as relevant case studies (Figure 1). Subject matter expert (clinical informatics, medical, allied health, nursing) and health consumer co-author perspectives formed an analysis of research trends, ethical considerations, and future directions that was grounded in the consumer experience.

## 3. Public Health Policy and Practice

### 3.1. Methods

Public health policy and practice relates to health promotion and disease prevention activities conducted at the population level. Precision public health is a modern iteration of traditional public health that leverages real-world data, including data from CHI, to precisely inform multi-level policy, interventions, and decisions to improve population health outcomes [ 13 , 14 ]. Real-world data encompasses health information routinely collected from sources outside traditional research and primary data collection settings, such as EHRs, mHealth, wearables, IoT, social media, genomics, and commercial transactions data [ 13 ]. Methods of population health surveillance predominantly link EHRs and health insurance providers with “traditional” data from Census or large population health surveys [ 13 ]. EHRs are an attractive population health surveillance tool due to the collection of routine and clinically measured health data at scale, and have emerged as a data foundation for guiding precision public health in Australia [ 8 , 15 ], Canada [ 16 ], China [ 17 ], and the USA [ 8 , 18 ]. Human genomics research is advancing precision public health, with examples of polygenic risk-tailored screening for breast, colorectal, prostate, and skin cancers [ 14 ], identifying rare diseases in newborn screening [ 19 ], and pharmacogenomics to reduce health inequalities [ 14 ]. Real-world data delivers scale that can guide CHI from single consumer-facing interventions to population-wide interventions. Just-in-time adaptive interventions (JITAIs) are one intervention example that can use individual-level data via multiple wearable sensors, such as accelerometers and GPS, or continuous streams of routinely collected population-based data to identify opportunistic intervention timepoints for behavioural change [ 20 ].

### 3.2. Intervention Case Studies

#### 3.2.1. Population Dietary Surveillance

Supermarket electronic purchase records can enable longitudinal dietary surveillance in high- and middle-income populations and guide a range of consumer purchasing, business, and community interventions which have supportive evidence for influencing purchasing behaviours [ 21 ]. Nudge-style interventions for consumer purchasing offer promise to influence food behaviour choices in real-time; although, evidence for effectiveness is weak [ 22 ].

#### 3.2.2. Physical Activity

Physical activity is an ideal target behaviour for JITAIs as they can intervene in response to changing contexts, environments, and behaviour that are measured by the quantified self (mHealth, wearables) [ 23 ]. A systematic review of the effectiveness of JITAIs to promote physical activity found mixed evidence for intervention effects on physical activity behaviour; but studies were insufficiently powered to detect behavioural changes [ 23 ]. Overall, JITAI's were acceptable to participants as an intervention modality [ 23 ].

#### 3.2.3. Genomics Screening

One example from human genomics analysed data from 409,258 participants in the UK Biobank to develop and validate genome-wide polygenic risk scores (PRS). These scores identified 8%, 6.1%, 3.5%, 3.2%, and 1.5% of the population at more than threefold increased risk for coronary artery disease, atrial fibrillation, type 2 diabetes, inflammatory bowel disease, and breast cancer, respectively[ 24 ]. PRS scores have clinical utility to inform more personalised interventions and disease screening pathways, and improve intervention cost-effectiveness at a population level [ 25 ]. In a simulated cohort of 4.48 million men, offering precision (risk-tailored) screening based on a PRS for developing prostate cancer could reduce overdiagnosis, maintain similar mortality benefits to age-based screening, and improve cost-effectiveness [ 26 ]. Similarly, providing consumers with personalised risk profiles for skin cancer (melanoma) development may motivate targeted prevention behaviours improve sun-related behaviours [ 27 ]. The ‘Melanoma Genomics Managing Your Risk’ study was a large ( *n* =948), population-based RCT that measured the impact of a personalised melanoma risk assessment via a polygenic risk score on outcomes of sun exposure, protection, and early detection behaviours. Genomic risk intervention did not affect ultraviolet exposure at 12 months; however, did reduce sunburns (RR 0.72, 0.54-0.96), improve skin examinations in women, and reduce melanoma-related stress [ 27 ], providing preliminary evidence for the potential effectiveness of precision prevention melanoma prevention strategies at the population level. Another study in young adults ( *n* =92) explored the impacts of personalized ultraviolet (UV) radiation photographs, genetic testing *(MC1R)* for skin cancer risk, and general skin cancer prevention education on sun-related behaviours (protection, tanning, sunburn) [ 28 ]. Providing a UV photo with a *MCR1* test resulted in a significant increase in avoidance of peak UV exposure (0.80 mean improvement) and a *MCR1* test alone was associated with significantly decreased outdoor unintentional tanning [ 28 ]. However, only receiving the UV photo increased sunburn occurrence post-intervention, and there were no observed between-group differences, indicating mixed results [ 28 ].

## 4. Individualized Disease Risk Assessment

### 4.1. Methods

Digital technologies facilitate the collection, integration, and analysis of vast real-world data via genomics, environmental factors, and behavioural choices to identify disease-specific risk factors [ 24 ]. Descriptive analytics can aggregate and display data to create ‘clinical intelligence’ for intervention design [ 29 ]. Predictive analytics in healthcare are supported by traditional regression methods and, more recently, powerful machine learning (ML) approaches to improve task speed and handle high-dimensional and unstructured healthcare data ( *e.g.* , from electronic medical records or medical images) for disease diagnosis or prognosis, and to estimate individualized treatment responses [ 30 , 31 ]. Traditional regression and ML methods leverage historical data to identify patterns and make accurate predictions, aiding in the development of personalized prevention strategies [ 32 , 33 ], although there is no clear performance benefit of ML over regression methods [ 34 ]. Clinical decision-support tools can then guide risk-based individualized treatment recommendations at the point-of-care [ 30 ].

Academic discourse has encouraged movement away from developing new prediction models and focusing on external and independent prospective model validations prior to ethical and methodologically robust impact evaluations of clinical prediction pathways on healthcare outcomes and health service delivery [ 35 ]. A recent development was SALIENT-AI, an evidence-based framework to guide the levels of evidence generation required to justify clinical deployment of AI algorithms in healthcare [ 36 , 37 ]. SALIENT-AI proposes the need to continuously update clinically implemented algorithms to monitor and track data shift, emergent algorithmic bias, and prediction quality [ 36 ] to move clinical prediction models from ‘set and forget’ to ‘set and reset’.

### 4.2. Intervention Case Studies

#### 4.2.1. Cardiovascular Disease

Identifying individuals who are at increased risk of developing cardiovascular disease (CVD) is necessary to guide targeted early intervention care. There are a variety of clinical prediction models for CVD, such as the Systematic Coronary Risk Evaluation (SCORE) [ 38 ], QRISK3 [ 39 ], and the World Health Organization cardiovascular disease risk charts [ 40 ], with clinical practice guidelines recommending CVD risk assessment as part of routine primary prevention care [ 41 ]. SCORE and QRISK3 (both developed using proportional hazards models) are freely available as CHI tools in the public domain to facilitate consumer-led risk assessment of CVD risk. One prospective study integrated non-contrast CT images for coronary calcium scoring with clinical parameters to train explainable ML models (extreme gradient boosting [XGBoost]) to predict myocardial infarction and cardiac death with good predictive accuracy over standard clinical risk assessment [ 42 ]. The evidence for CVD risk assessment to improve the primary prevention of cardiovascular disease is mostly absent, with indications that total risk assessments have no effect on fatal and non-fatal cardiovascular events compared to standard care; however, are safe to implement and may slightly reduce risk factors such as blood pressure, cholesterol, and smoking levels in high-risk patient groups [ 41 ].

#### 4.2.2. Obesity prediction

Measuring individualised risk of developing childhood overweight and obesity can be achieved with simple, routinely available health and demographic data at the consumer-level ( *e.g.* , infant weight gain, maternal pre-pregnancy BMI [ 43 ]) and is a potential therapeutic intervention for obesity prevention [ 43 44 45 46 47 ]. A meta-analysis of prediction model performance (including logistic regression and ML methods *e.g.* , random forest, XGBoost) to predict overweight and obesity in children and adolescents found an overall pooled c-index of 0.835 (95% CI 0.792-0.879) and 0.769 (95% CI 0.754-0.785), indicating the models' pooled ability to discriminate between children who would go on to develop overweight or obesity with 83.5% and 76.9% accuracy, respectively [ 48 ]. Models developed using XGBoost (pooled c-index 0.810, 95% CI 0.809-0.811) appeared to perform better than logistic regression (pooled c-index 0.786, 95% CI 0.752-0.820); however, less data was analysed ( *n* =3 models versus *n* =20 models, respectively) [ 48 ]. Prediction models for childhood overweight/obesity have been developed globally in countries such as the US, China, UK, Malaysia, South Korea, Greece, Israel, and Australia[ 48 ], with two examples in the UK (SLOPE CORE and ProAsk) progressing to pilot feasibility testing in clinical practice as an interventional pathway for childhood obesity prevention [ 49 , 50 ].

## 5. Early Detection and Monitoring

### 5.1. Methods

Continuous monitoring through mHealth applications, wearable devices and health sensors enables the (near) real-time tracking of physiological parameters. For example, wearable devices offer continuous data streams of vital signs and health behaviours that enable precise health assessments and could be used to pre-empt disease exacerbation or hospitalisation once algorithms for early detection are developed and prospectively validated [ 51 ]. This allows for the early detection of deviations from baseline health, facilitating timely intervention and preventive measures. This method of continuous detection and monitoring contrasts with individualised disease risk assessment that learns predictor-outcome relationships in cross-sectional or retrospective data that is collected via specialised research methods or physiological testing. Digital health enables the “quantified self” where consumers use mHealth and wearable technologies to routinely collect and view personal data - their *digital phenotype* [ 52 ]. This digital phenotype informs individualised health decisions (at the consumer level) and interventions (at the provider level) across progressive stages of engagement from simple ‘collection’ to complex ‘integration’ and ‘action’[ 53 ]. Digital phenotype data can include traditional biomarkers such as heart rate variability and blood pressure but can now extend to measures of mental health ( *e.g.* , stress) and glycaemic control ( *e.g.* , continuous blood glucose monitoring). These data can be directly acted upon by the consumer without health practitioner support *e.g.* , varying diet according to blood glucose levels. This reduces the need for contact with the healthcare system and can create precise and real time adjustments to lifestyle to avoid disease and its complications.

### 5.2. Intervention Case Studies

#### 5.2.1. Cardiovascular Disease

A systematic review and meta-analysis [ 54 ] examined the effectiveness of digital health interventions on CVD risk scores in patients with increased CVD risk compared to standard care and found no effect for digital health interventions compared to standard care (moderate evidence); however, automated email messaging with a complementary web application was the most effective digital intervention component and had a potential protective effect [ 54 ]. The authors cited an overall lack of trial evidence to support a comprehensive evaluation of effectiveness, with further trials warranted in wearable devices that continuously record CVD-related data ( *e.g.* , blood pressure, heart rate) [ 54 ]. The CONNECT multi-centre RCT tested the effect of an interactive mHealth app on preventive behaviours in 934 patients at-risk of CVD in primary health care and found some improvement in blood pressure and LDL cholesterol, and significant improvements in physical activity targets and e-health literacy scores, but was not effective at improving medication adherence [ 55 ]. In the Apple Heart Study ( *n* =419,297), a smartwatch that routinely collected pulse wave data was tested for its ability to identify atrial fibrillation (AF), and correctly identified participants who received an irregular pulse notification with observed AF via electrocardiography 84% of the time (positive predictive value 0.84, 95% CI 0.76-0.92), supporting smartwatch-enabled detection of AF at scale [ 56 ].

#### 5.2.2. Smoking Relapse

Sense2Stop is a planned micro-randomized trial [ 57 ] to use wearable devices to tailor JITAI stress management intervention to promote smoking relapse prevention. A pilot feasibility JITAI intervention that delivered brief mindfulness and motivational strategies via smartphone in real-time based on a negative stress reading or detected smoking found high retention (83.7%) and treatment satisfaction with moderate intervention adherence [ 58 ]. Real-time detection of human behaviour and physiological responses to high-risk relapse scenarios could improve the precision of decision rules designed to deliver JITAIs for smoking relapse prevention [ 58 ].

## 6. Tailored Interventions of Modifiable Health Determinants

### 6.1. Methods

Digital health platforms ( *e.g.* , mHealth, wearables) offer providers the ability to provide personalized recommendations for behavioural change (dietary, physical activity, sleep) based on individual health data that have shown preliminary effectiveness in improving behavioural health outcomes [ 59 ]. A tailored approach that is supported by automated data collection potentially increases the likelihood of individuals adopting and sustaining healthy behaviours [ 59 ]. Providers can access and review consumer health data and devise preventive interventions that are precisely tailored to the individual's circumstances [ 60 ]. Topol (2019) argued that artificial intelligence (AI) is a catalyst for the promise of “high-performance medicine”, whereby AI can quickly condense massive and heterogeneous datasets into clinically actionable intelligence [ 51 ]; however, the same vision can be applied to consumers and “high-performance health”, with AI driving (near) real-time synthesis of consumer health information to offer actions related to modifiable health behaviours. Many smartwatches already offer digital ‘nudges’ when sedentary behaviour is detected *e.g.* , the Apple Watch includes a “Time to Stand” nudge when it detects a period of sitting for the first 50 minutes of an hour that can increase the probability of standing by up to 43.9% that maintains effectiveness over time [ 61 ].

Causal ML is a branch of AI that leverages causal reasoning to estimate the individualised effect of a treatment decision [ 30 , 62 ]. By estimating a causal quantity, such as the conditional average treatment effect ( *i.e.* , the treatment effect for a specific sub-population), causal ML can predict changes in patient outcomes due to various treatment options, thus offering personalized predictions of treatment efficacy that can advance precision healthcare. Causal relationships are traditionally learned through randomized controlled trials. However, ML can analyze and integrate high-dimensional observational data from EHRs and other real-world data sources, such as genomics, to estimate causal effects [ 62 , 63 ]. For example, by integrating personal pharmacogenomics and clinical data, causal ML was used to accurately predict individual responses to serotonin reuptake inhibitor remission and response in patients with major depressive disorder with discriminative accuracy (AUC) of >70% [ 64 ]. By offering personalized predictions of intervention efficacy, causal ML can potentially advance precision care across the prevention continuum; however, most efficacy studies have been conducted in simulated (shadow) clinical data rather than real-world settings.

### 6.2. Intervention Case Studies

#### 6.2.1. Precision Nutrition

The PREDICT 1 study recruited 1,002 twins from the TwinsUK cohort and found substantial between-individual variability in metabolic postprandial responses to triglycerides, glucose, and insulin following identical meals that was explained by heterogeneity in individual gut microbiome composition and genetic variants [ 65 ]. A ML model was developed to predict individual response to triglyceride and glycaemic food responses. Participants engaged with a mHealth app (‘ZOE’) to digitally record study adherence, dietary intake, and physical activity, and to communicate with the study team. Informed by these results, the ZOE METHOD open-label RCT found a personalized dietary program based upon individual postprandial food responses, microbiomes, and health indicators improved triglycerides and other outcome measures ( *e.g.* , body weight, HbA1c, diet quality) but did not improve low-density lipoprotein cholesterol and other outcomes ( *e.g.* , blood pressure, glucose, postprandial triglycerides) compared to standard dietary advice [ 66 ]. Another RCT (Personal Diet Study) evaluated a precision nutrition diet to improve weight loss in adults with abnormal glucose metabolism and obesity compared to a standardized low fat diet and did not find significant between-group differences in weight loss [ 67 ].

#### 6.2.2. Childhood Obesity

A Childhood Obesity Risk Estimation tool (SLOPE CORE) [ 49 ] was developed using externally-validated prediction models of routinely available maternity and early childhood health data [ 47 , 68 , 69 ] in the UK to calculate the risk of childhood obesity (ages 4-5 years) and provide health professionals with a potential digital interventional pathway for early childhood obesity prevention. SLOPE CORE was evaluated in a small feasibility study with five health visitor's and seven parents, with results indicating high usability of the tool in practice and acceptability within their sample of parents [ 49 ]. A similar prediction model for childhood obesity, ProAsk [ 50 ], was evaluated in a multi-centre pre-post intervention feasibility study in UK community care within rural and urban deprived settings. ProAsk was delivered by 22 health visitors to 66 parents via digital technology to quantify an infant's likelihood of developing future obesity; however, while ProAsk was acceptable to most parents and health visitors, fidelity to ProAsk interventions was low, and some health visitors were uncertain about communicating obesity risk to parents of infants [ 50 ]. There is general optimism for the utility of clinical prediction models as a preventive pathway for childhood obesity; however, barriers related to intervention effectiveness, navigating potentially stigmatising and harmful conversations, and challenging traditional cognitive and motivational biases persist [ 70 ]. In the TOPCHILD collaboration, a multinational individual participant data meta-analysis will systematically map intervention components for early childhood obesity prevention interventions and evaluate intervention effects across individual demographic characteristics [ 71 ]. Prediction models will be developed to understand which individual, or group of individuals, is more likely to benefit from any given intervention component to potentially inform precision prevention pathways for childhood obesity that are optimised for treatment effectiveness [ 72 ].

## 7. Research Trends, Ethical Considerations, and Future Directions

Current research trends on the topic of CHI for precision prevention extend the person-centred care and healthcare quality improvement discourse of the recent few decades. Person- and population-centred precision prevention relies on quality, representative person and population level data shared with healthcare services for analysis in an agile and responsive format [ 72 73 74 ]. This format, however, requires a shift in thinking away from illness-orientation and towards a partnership of shared data, equal power and an expanded role of the consumer as a specialist in lived experience and observation [ 29 ]. We propose that CHI for precision prevention will be enabled by two streams of work: empowered consumers and communities; and an enabling healthcare environment. Partnerships among consumers and providers redistribute power more evenly and generate research and practice benefits proportional to the strength of investment in iterative, meaningful “push” from consumers and communities and “pull” from providers. The two streams of work are discussed below in terms of research trends, ethical considerations and possible future directions.

### 7.1. Empowered Consumers and Communities

Research discourse on empowered consumers and communities has recently focused on three aspects. First, digital health literacy of consumers is receiving growing attention, believed to comprise of information literacy, computer literacy, digital literacy and health literacy [ 29 ]. Consumer digital health literacy provides the foundational skills to enable a more active role in the decision-making process when using primordial, primary, and secondary prevention interventions to improve health [ 75 ]. Education and targeted design through collaborative efforts are needed to address the variability of digital health literacy in populations and older, younger, and socio-economically or geographically disadvantaged groups are particularly at risk [ 76 ].

Second, CHI intervention methodologies are extending beyond considering cultural norms and values of traditionally researched groups [ 77 ] to consider design requirements of all population groups to enhance the consumer experience [ 78 ]. An increased focus on inclusive design methodologies [ 78 ] are observed in the academic literature, providing researchers and practitioners with on guidance of cultural tailoring of CHI interventions for content, functionality, interface and platform relevant to minority groups [ 77 ].

Third, precision prevention assumes the safe use of large datasets but in the context of data breaches and commercialisation conflicts of interest, questions have been raised about the caveats of this secondary use of health data. Ethical governance outside the approval of a hospital or research study (consented data or consent waiver) to the general public over time requires critical analysis across concepts such as privacy, risk, trust and transparency [ 79 ]. The expectations of society regarding the conduct of data use may be referred to as a social licence; a concept that is increasingly being explored but the theoretical and practical implications are still largely unknown [ 79 ] and remains an important research priority.

### 7.2. Enabling Healthcare Environment

Research evidence to create an enabling healthcare environment that is accepting of consumer data for precision prevention is emerging. First, the role that individuals play within the health and wellbeing system is developing. The goal of patient-centred care is for a *functional* life, to now the goal of person-centred care is for a *meaningful* life [ 80 ]. This perceivably small but significant change in language elicits a response for systems to consider people's wider social and cultural needs, capabilities, and resources [ 80 , 81 ] and a focus towards prevention healthcare.

Second, community engagement approaches for CHI design have matured in recent years. Co-design has become increasingly pervasive over the last decade [ 29 ]. However, its use in practice is inconsistent and generates variable success. There are criticisms that co-design can suffer from prolonged timelines, poor impact evaluations, and tokenism [ 82 83 84 ]. For co-design to be successful and measurable, principles are being formalised, for example the Social Care Institute for Excellence in the UK articulate four critical values of equality, diversity, accessibility and reciprocity [ 84 ]. Approaches which provide the opportunity to contribute data from different health settings and online settings ( *e.g.* , crowdsourcing, citizen science) or beyond traditional health service users to broader range of stakeholders ( *e.g.* , World Cafe, Citizens Assembly) [ 29 ] are emerging. Novel methods to represent experience data in a digital context ( *e.g.* , persona use cases, digital twins) are being borrowed from engineering domains and applied to health [ 52 ]. The challenge now is for future research to determine how to best use the collective experience of various individuals to benefit whole communities and practitioners [ 52 ].

Third, the technology is advancing in support of health services learning from disparate datasets. Learning from datasets beyond traditional EHR data to placed-based data collected in communities ( *e.g.* , churches, tribal areas, community meetings) will be more representative of consumers and their population groups; this aims to avoid aggravating health disparities in chronic disease risk among racial and ethnic minorities and other vulnerable populations [ 85 ] that might not generate longitudinal EHR data due to access issues. This will be a key tenet to designing precision public health interventions that reflect real-world and representative data from diverse sources, such as nutrition policy informed by aggregated supermarket purchase data, cultural nutrition beliefs and practices, dietary surveys, and food access and security. As data remains in silos when generated on disparate CHI systems, novel technical solutions are needed to share learnings across datasets while respecting local data governance requirements [ 86 ]. Federated learning is one such ‘privacy-preserving’ approach [ 86 ] and has been explored in mHealth [ 87 ] and rare disease registry hubs [ 88 ], but remains focused on remote monitoring, diagnostic and treatment support [ 87 ] or connecting groups of people [ 88 ] respectively, falling short of precision prevention.

## 8. Conclusion

CHI is a transformative agent in healthcare and public health, offering a data-driven opportunity to improve the effectiveness of care across the prevention continuum. In the intervention case studies presented, CHI demonstrated mixed evidence for effectiveness in improving risk-stratified or behavioural prevention-related health outcomes for chronic disease. As the field continues to evolve, collaborative efforts among consumers, populations, researchers, healthcare professionals, and policymakers are essential to design and conduct methodologically robust studies that generate high-quality evidence for effectiveness to prevent or delay the onset of disease in populations with the highest risk and need.

## Notes

Box 1: Synthesised definition of precision prevention Tailored interventions to improve one or more modifiable biological, social, environmental, or behavioural characteristic of an individual or population to reduce disease risk or delay the onset of disease.
